# Regional Variation in Causes of Injuries among Terrorism Victims for Mass Casualty Events

**DOI:** 10.3389/fpubh.2015.00198

**Published:** 2015-08-17

**Authors:** James L. Regens, Amy Schultheiss, Nick Mould

**Affiliations:** ^1^OU Center for Intelligence and National Security, University of Oklahoma Health Sciences Center, Oklahoma City, OK, USA

**Keywords:** terrorism, mass casualty incidents, injury patterns, regional variation, disasters

## Abstract

The efficient allocation of medical resources to prepare for and respond to mass casualty events (MCEs) attributable to intentional acts of terrorism is a major challenge confronting disaster planners and emergency personnel. This research article examines variation in regional patterns in the causes of injures associated with 77,258 successful terrorist attacks that occurred between 1970 and 2013 involving the use of explosives, firearms, and/or incendiaries. The objective of this research is to estimate regional variation in the use of different conventional weapons in successful terrorist attacks in each world region on variation in injury cause distributions. Indeed, we find that the distributions of the number of injuries attributable to specific weapons types (i.e., by cause) vary greatly among the 13 world regions identified within the Global Terrorism Database.

## Introduction

Although deaths due to terrorism unlike traffic accidents account for a relatively small percentage of trauma deaths annually, nonetheless, successful terrorist attacks have become an increasingly common phenomenon since 1970s. On a global scale, approximately 125,000 attacks potentially involving terrorism occurred between 1970 and 2013 (1). With the exception of Antarctica, no continent has been spared from experiencing intentional violent acts that are designed to affect the behavior of targeted populations and governments by producing widespread deaths and injuries. Examples include Amman, Boston, Buenos Aires, London, Mumbai, New York, Nairobi, Oklahoma City, Paris, Tel Aviv, and Tokyo. Analysis of patterns in the use of conventional weapons and tactics in each world region can contribute to developing a realistic appreciation of variation in injury cause distributions among those regions because different types of weapons produce different kinds of injuries.

Similar to warfare (2, 3), mass casualty events (MCEs) due to terrorist attacks are known to produce distinct injuries when compared to other major causes of injuries, such as automobile accidents or traditional criminal violence. These unique injury causes associated with terrorism pose significant challenges to existing emergency response systems (4–9). An MCE is defined as an event, sometimes called a multiple-casualty incident or multiple-casualty situation, in which the number and severity of injuries exceeding the normal capacity overwhelm emergency medical services resources associated with those resources (10). Although a natural or human-induced disaster by definition overwhelms response capabilities, a mass casualty incident (MCI) occurs more commonly and is defined as a situation that places a significant demand on medical resources and personnel. Local response capabilities are not overwhelmed, but there are still a large number of patients requiring triage (11).

Empirical evidence indicates that the *severity* of non-lethal injuries is a function of the type of weapon utilized in the attack while the *magnitude* of a MCE is a function of the number of people injured or killed by the attack (1). Explosions, for example, cause more complex and multiple forms of damage than other conventional weapons types that produce wounds primarily due to blast and crush effects, including traumatic amputation of extremities, ruptured eardrums, mild to severe traumatic brain injury, and/or penetrating injuries from shrapnel. Burns and gunshot wounds account for the majority of the remaining casualties from terrorism. Incendiary devices cause thermal injuries to skin tissue ranging from superficial first degree burns through severe third degree burns with the amount of damage caused due to a burn being dependent upon its location, its depth, and the amount of body surface area affected. Non-lethal ballistic wounds due to the use of firearms, commonly referred to as gunshot wounds, involve penetrating injuries, such as damage to tissue and organs, severe bleeding, broken bones, and/or paralysis when a bullet or other projectile penetrates the body with the severity of damage a function of the projectile’s velocity and mass and the locus of the injury. Thus, when viewed from a disaster medicine perspective, it is essential to be able to anticipate the types of weapons that are likely to be associated with injury patterns for terrorist-centric MCEs in order to prepare for and respond quickly as well as effectively to those events. In essence, collecting and analyzing information about the causes of injury patterns among terrorism victims who are not killed by those attacks can provide the understanding needed to design and implement strategies for coping adequately with these types of disaster medicine and public health challenges.

In this article, we examine regional variation in the causes of injury patterns among terrorism victims for mass casualty events between 1970 and 2013 inclusive based on the predominant types of weapons used for all successful attacks that were reported in the open source literature during that time period. Drawing on those historical data, we consider the resulting distribution of injuries by weapon type and major geographical region of the world to determine expected injuries per region.

In this analysis, we exclude all deaths *in situ* related to the initial event. Although the total number of individuals killed at the scene of the attack is important for preparing and allocating mortuary services or for comparing deaths from terrorism to other causes of mortality (12–14), we opt to exclude the number killed at the locus of the attack because preparedness for comparing deaths from terrorism to other causes of deaths *per se* is not analogous to preparedness for MCE trauma injuries even though both outcomes may stem from the same proximate cause (i.e., weapon and tactic employed in the attack). We realize that a portion of the critical injuries received by immediate survivors may have eventually led to “late” death thereby increasing the overall mortality from the attack. However, those *non-in situ* deaths are treated as injuries in the database, and the ultimate outcome with respect to deaths away from the scene of the attack is unknown. Moreover, the number killed versus those injured in an attack is, in part, a function of the interplay of a variety of factors, such as the weapon type (e.g., a bullet from a firearm typically results in a single casualty/bullet versus multiple casualties from a bomb), the environmental setting (e.g., a confined space amplifies the ballast wave propagated by an explosive while an open space facilitates dissipation), anatomic sites of injury, accurate triage, elapsed time between injury and treatment, and/or the specific target (e.g., a bus, nightclub, market, individual, etc.). Focusing exclusively on injury totals associated with individual attacks allows us to identify convergence and divergence in terms of the causes of injury patterns. Our hypothesis is that the distributions for the causes of injuries will vary significantly among world regions as a function of weapons. Delineating underlying patterns in the data can illuminate similar regions and, based on the likely weapons and tactics predominately employed in a specific region, help elucidate resource needs for the medical management of MCEs from terrorism because specific kinds of injuries are associated with specific types of weapons (15–17).

## Materials and Methods

We use data from the global terrorism database (GTD), an open source database, which contains information on approximately 125,000 attacks that potentially constituted acts of terrorism between 1970 and 2013 (1). The records in the GTD are compiled from media reporting of individual attacks. The GTD is the largest and most comprehensive publically available dataset available to researchers. The observations (i.e., individual attacks) come from all regions, which makes geographically diverse comparisons possible, rather than being limited to a single country or region. Moreover, it covers a 43-year timeframe that captures the contemporary era of terrorism. Because the data are compiled from media reports, we note that this potentially may result in some underreporting of the total number of successful attacks with those events most likely to have happened in countries that restrict freedom of the press. However, any underreporting is likely to be relatively modest in the aggregate given the diffusion of social media, the Internet, and cellular telephones since 1990s. As a result, we maintain that the shear size of the database combined with the fact that it includes incidents spanning almost 45 years and multiple countries capturing the modern era of terrorism outweighs any drawback attributable to possible gaps in the database. A critical analysis of the GTD data should allow identification of underlying patterns in causes of injuries among terrorism victims.

Table 1 summarizes the primary weapons responsible for the majority of injuries in the GTD. The explosives (other) category is used to characterize all explosion-related attacks, such as the use of grenades that did not involve a vehicle or a suicide. For each weapon type, the number of injuries per attack is also provided. The five weapon types identified in Table 1 constitute 91.7% of the 77,258 successful attacks and 93.2% of the 302,275 injuries reported in the GTD (1). Hence, it is reasonable to conclude that these weapons types predominate in successful, or for that matter, failed terrorist attacks. In these calculations, we excluded events where the number of injuries was unknown or the likelihood of terrorism was in doubt. Because the overwhelming majority of successful terrorist attacks are based on the use of explosives, firearms, and incendiary devices, the importance of understanding the relationships between attack types, and by extension the expected injury modes that are likely to require specialized medical care, cannot be overstated (1).

**Table 1:** **Prominent weapon types and the expected number of injuries per event (1970–2013)**.

Weapon type	Percentage of attacks	Injuries per event
Explosives (other) (*n* = 37,233)	48.19	3.79
Firearms (*n* = 23,985)	31.05	1.57
Incendiary (*n* = 4,751)	6.15	0.66
Explosives (vehicle) (*n* = 3,675)	4.76	18.82
Explosives (suicide) (*n* = 1,219)	1.58	25.04

In order to delineate regional variation in the causes of injury patterns among terrorism victims for MCEs, we consider the distribution of injuries per world region over the five prominent weapon types previously identified in Table 1. The GTD groups attacks into 13 different world regions based on conventional definitions of region used in geography: (1) North America, (2) Central America and Caribbean, (3) South America, (4) East Asia, (5) Southeast Asia, (6) South Asia, (7) Central Asia, (8) Western Europe, (1) Eastern Europe, (10) Middle East and North Africa, (11) Sub-Saharan Africa, (12) Russia and the Newly Independent States (NIS), and (13) Australasia and Oceania. For each region, we estimate the distribution of injuries across the different weapon types over the time period beginning in 1970 and ending in 2013, with the caveat that data from 1993 are unavailable. These results in 13 region-specific injury distributions that characterize the expected distribution of injuries associated with an arbitrary terrorist attack. We use the term “estimate” to refer to these distributions because it conceivable as noted above to assume that a small but non-quantifiable number of events are not captured in the GTD. It is prudent, therefore, to explain to prospective readers who may not be sophisticated statistically that the computed results are estimates albeit values derived from a very large database that is likely to encompass the overwhelming total of all successful terrorist attacks globally during the time period cover by this analysis.

To estimate each regional MCE injury distribution attributable to successful terrorist attacks, we queried the GTD for all events using a series of filters to screen out those incidents that do not unambiguously involve terrorism: (1) the violent act must be aimed at attaining a political, economic, religious, or social goal; (2) there must be evidence of an intention to coerce, intimidate, or convey some other message to a larger audience (or audiences) than the immediate victims; and (3) the action is outside the context of legitimate warfare activities, insofar as it targetsnon combatants(i.e., the act must be outside the parameters permitted by international humanitarian law as reflected in the Additional Protocol to the Geneva Conventions of 12 August 1949 and elsewhere). The GTD also incorporates a “doubt factor” for inclusion of otherwise ambiguous cases for which there is essentially no doubt as to whether the incident is an act of terrorism. The application of these initial criteria results in an *n* = 84,069 successful terrorist attacks that meet the above criteria.

We then evaluate whether each of the events met two conditions. First, the weapon type used by the perpetrators of the terrorist attack was known to be explosives (suicide, vehicle, or other), firearms, or incendiaries. Second, both the region and the number of victims injured by the attack were known. This filtering produces 77,258 observations which meet both conditions. All of these events were then grouped by region and weapon type, resulting in 13 × 5 = 65 tabular datasets, where each row of each table contained the number of people injured associated with each terrorist attack. Finally, the total number of injuries associated with each successful attack was grouped into five element vectors by direct summation, where each element corresponds to a specific weapon type, and normalized to produce an estimate of the MCE injuries in each world region.

## Results

Our analysis of variation in the regional distributions for the causes of MCE injuries for victims of terrorist attacks is based on a total of 77,258 terrorist attacks. The weapon type(s) consistent with Table 2, the geographic region, and the number of victims injured are known for each of those incidents that unambiguously met the criteria outlined above for being defined as involving deliberate acts of terrorism.

**Table 2:** **Regional MCE injury distributions grouped by cluster**.

Region	Distribution of injuries				
	Firearms	Explosives (suicide)	Explosives (vehicle)	Explosives (other)	Incendiary
Cluster center 1	0.20	0.07	0.25	0.46	0.03
Middle East and North Africa (*n* = 17,307)	0.06	0.14	0.40	0.39	0.00
Sub-Saharan Africa (*n* = 4,796)	0.29	0.01	0.24	0.44	0.02
Russia and the newly independent states (NIS) (*n* = 1,502)	0.20	0.11	0.21	0.48	0.00
Australasia and Oceania (*n* = 74)	0.24	0.00	0.15	0.52	0.09
Cluster center 2	0.15	0.04	0.07	0.67	0.07
North America (*n* = 1,649)	0.13	0.00	0.01	0.73	0.13
South America (*n* = 10,264)	0.22	0.00	0.12	0.64	0.01
East Asia (*n* = 260)	0.07	0.03	0.03	0.60	0.26
Southeast Asia (*n* = 5,245)	0.18	0.04	0.10	0.66	0.02
South Asia (*n* = 17,739)	0.15	0.13	0.13	0.58	0.01
Western Europe (*n* = 7,638)	0.10	0.10	0.10	0.72	0.03
Eastern Europe (*n* = 542)	0.17	0.02	0.02	0.78	0.01
Cluster center 3	0.61	0.01	0.01	0.35	0.01
Central America and Caribbean (*n* = 3,730)	0.50	0.00	0.00	0.48	0.01
Central Asia (*n* = 117)	0.72	0.02	0.02	0.22	0.00

**Figure 1:**
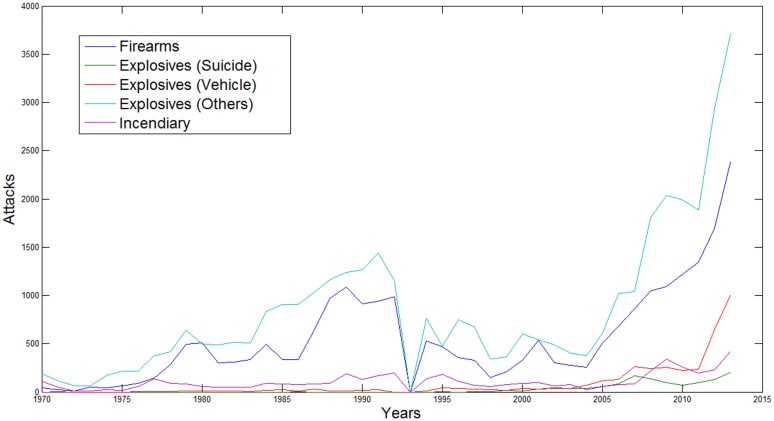
**Number of attacks per year grouped by weapon type (1970–2013)**.

Figure 1 illustrates the number of attacks by weapon type over the time period 1970–2013. Note that data for 1993 are not available. Not surprisingly, the patterns in the data reveal that explosives other than suicide bombings and vehicle-borne improvised explosive devices (e.g., mail bombs, dynamite/TNT, and other explosives) and firearms consistently have been the dominant weapon types employed in terrorist attacks. Reliance on suicide bombings, incendiary devices, and vehicle-borne improvised explosive devices (VBIEDs) – especially VBIEDs – increased substantially after 2001.

Table 2 depicts the estimated MCE injury distributions for each of the thirteen world regions. Each row in Table 2 corresponds to the distribution of attacks by weapon type for a particular world region. Values are summed across an individual row to determine the percentage of injuries by weapon type that occurred within a region. The values within each row should sum to unity with the exception of small (1–2%) variations due to rounding. For example, the row for the Middle East and North Africa indicates that 20% of the injuries were attributable to firearms, 7% to suicide bombings, 25% to VBIEDs, 46% to explosives other than suicide bombings or VBIEDs, and 3% of the injury pattern stemmed from incendiary devices. Examination of each cell down a column (i.e., weapon type) reveals differentials in the distribution of injuries for that specific weapon type. For example, as noted above, firearms accounted for 20% of the injuries from successful terrorist attacks in the Middle East and North Africa, 10% in Western Europe, and 72% in Central Asia. The variation among distributions of injury causes reflects differences in the tactics, techniques, and procedures employed by terrorists among regions.

**Figure 2:**
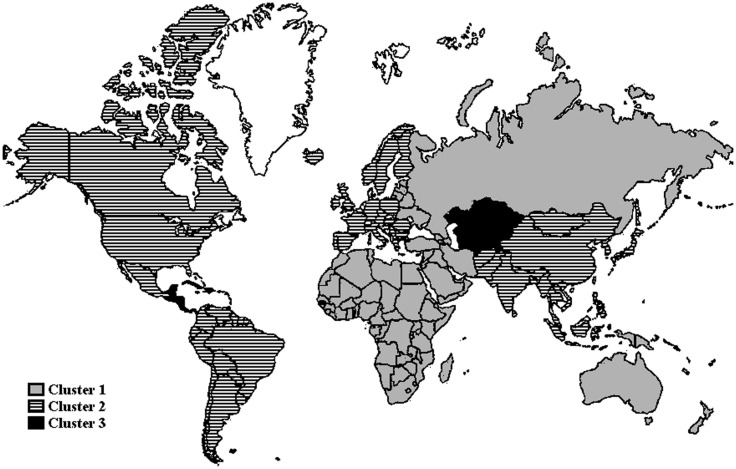
**Geographical illustration of regional clusters identified in Table 2**.

In addition, we performed clustering on the MCE injury distributions using the k-means clustering algorithm for vector quantification (9, 18, 19). K-means clustering, originally used for machine learning, groups the observations into a set of distinct clusters in which each observation belongs to the cluster with the nearest mean which makes it possible to partition (i.e., cluster) the data space into Voronoi cells by partitioning a plane into regions based on the distance to points in a specific subset of the plane. Application of k-means clustering organized the data in Table 2 by grouping the distributions into three identified clusters of geographical regions. Figure 2 depicts the clusters geographically using the world region clustering shown in Table 2. The purpose of the clustering is to identify dense regions within the joint distribution of weapons, regions, and injuries. The purpose of identifying dense regions in any distribution is to locate high-probability regions in the sample space. In this specific joint distribution, the dense regions correspond to areas where specific weapon combinations cause large numbers of injuries.

Based on examination of the cluster centers, we observe that the estimated MCE injury distributions associated with each cluster vary significantly. Turning first to injury patterns tied to attacks involving firearms, the data reveal that variation exists within each cluster but that firearms are expected to cause 20% of the cluster 1 injuries, 15% of the cluster 2 injuries, and 61% of the cluster 3 injuries. Hence, victims of terrorist attacks happening in cluster 3 – especially incidents in Central America and the Caribbean – are at substantially greater risk of primarily experiencing ballistic injuries from firearms than are individuals in each of the other two clusters. On the other hand, attacks that involved suicide bombings, vehicle-borne improvised explosive devices, or incendiary devices were extremely rare events. Blast wave injuries and ballistic injuries tied to suicide bombings or vehicle-borne improvised explosive devices (VBIEDs) are most likely to occur in cluster 1.

## Discussion

In some cases, the geographical regions sharing similar injury patterns stemming from the types of weapons used in successful terrorist attacks are not surprising. For example, cluster 1 includes the Middle East and North Africa where suicide bombings or VBIEDs have been employed frequently coinciding in large measure with the second Intifada in Israel and Palestine, the expansion of the jihadist movement, and the insurgency in Iraq following the US led overthrow of Saddam Hussein (20). It is also not surprising that, perhaps reflecting broadly shared cultures and/or geographic proximity, cluster 2 includes North America, Western Europe, and Eastern Europe. Blast wave injuries and ballistic injuries tied to suicide bombings or vehicle-borne improvised explosive devices (VBIEDs) are most likely to occur in cluster 1. On the other hand, the regions associated with each cluster are in most cases are unexpected. The inclusion of sub-Saharan Africa and both Russia and Australasia and Oceania in cluster 1 or the grouping of Central Asia and Central America and Caribbean in cluster 3 exemplifies this.

Because unique injury causes and specialized medical care are known to be associated with each of the five weapon types, the causes for the regional MCE injury distributions provide powerful insights into the expected emergency medical needs in each of the 13 world regions. The ability to respond to MCEs involving ballistic injuries from the use of firearms tends to be common to all regions regardless of cluster underscoring the need for trauma surgical capabilities is a generic capacity. Blast wave injuries, particularly blast-induced neurotrauma (BINT), are less uniformly distributed within and across the clusters reflecting greater regional variation in the use of various types of explosives. This result suggests that investments in specialized neurotrauma resources are not a uniformly generic requirement. Similarly, as noted above, burn injuries from the use of incendiary devices are not the dominant injury pattern manifesting in terrorist attacks other than for East Asia.

We offer the caveat that, in terms of limitations, these causes of MCE injury distributions are estimated based on an extended over 40-year time period. Possible sensitivity to temporal effects could be addressed by estimation of MCE distributions for more recent or shorter time periods. Additionally, we are uncertain about the true number of clusters associated with the MCE injury distributions, but our estimates based on iterative clustering and random initial cluster centers suggest three clusters. It is well know that there is no method for determining the number of clusters of dense regions in statistical distributions. With the above caveat in mind, our analysis indicates that there are approximately 3 different MCE injury patterns associated with type of weapons used that occur over the 13 world regions examined in this study.

These three MCE injury clusters indicate that the emergency response needs due to terrorism vary significantly in terms of the injury causes. Irrespective of the clustering results, it is clear that MCE injury distributions vary greatly by geographical region. This research demonstrates that regional MCE distributions could be used directly to estimate the emergency medical needs per region instead of using a clustering algorithm. Furthermore, this approach could be extended beyond regions to the level of specific countries or even less aggregated, such as metropolitan areas in order to produce higher resolution (geographically specific) injury estimates.

Consequently, the identification of regional or for that matter sub-regional variation in the distribution of injury patterns among victims of terrorism is directly relevant to emergency medicine planning and response. The injuries associated with terrorism are likely to be critical and, therefore, expensive to address. In part, this reflects the fact that proximity spatially to the event rather than the total amount of resources available within a region or smaller geographical unit is likely to be a critical factor in providing prompt and effective clinical care immediately after a terrorist attack (i.e., the “golden hour”). Moreover, the stock of intensive care units, medical imaging technology (e.g., CT, functional MRI), burns units, and Level 1 trauma centers is not uniformly distributed but instead is limited spatially and temporally. We maintain this inevitably leads to recognition of the requirement to prioritize preparedness planning based on likelihood estimates grounded in empirical evidence for the types of injuries expected and the number of victims categorized by injury cause routinely probable to present at hospitals and clinics as well as for triage in the field. Simply put, if you know what types of conventional weapons used in successful terrorist attacks predominate in a region, then *ceteris paribus* you have an increased likelihood of anticipating the types of injuries that will manifest because specific injuries are caused by specific weapons. This study can help to inform that process.

## Conflict of Interest Statement

The authors declare that the research was conducted in the absence of any commercial or financial relationships that could be construed as a potential conflict of interest.
